# Optical chaotic signal recovery in turbulent environments using a programmable optical processor

**DOI:** 10.1038/s41377-025-01784-3

**Published:** 2025-03-21

**Authors:** Sara Zaminga, Andres Martinez, Heming Huang, Damien Rontani, Francesco Morichetti, Andrea Melloni, Frédéric Grillot

**Affiliations:** 1 LTCI Télécom Paris, Institut Polytechnique de Paris, Palaiseau, 91120 France; 2 Dipartimento di Elettronica, Informazione e Bioingegneria, Politecnico di Milano, Milano, 20133 Italy; 3 Chair in Photonics, LMOPS UR 4423 Lab, CentraleSupélec & Université de Lorraine, Metz, 57070 France; 4 Center for High Technology Materials, University of New-Mexico, Albuquerque, NM 87106 USA

**Keywords:** Semiconductor lasers, Atmospheric optics, Integrated optics, Silicon photonics, Fibre optics and optical communications

## Abstract

Optical chaos offers a promising approach to establishing secure communication at high data rates in a shared physical channel, like optical fibers and free space. However, the required synchronization between the transmitter and the receiver can be severely impaired by the nonidealities of the optical link. In particular, free-space optical communications are affected by atmospheric turbulence, which causes beam scintillation and results in time-varying fading of the optical intensity at the receiver side. In this work, we investigate experimentally the propagation of a chaotic signal in an indoor optical link with controllable synthetic turbulence, and we show that the degradation of chaos properties caused by the turbulent environment can be fully mitigated in the optical domain using an adaptive multi-aperture receiver. The proposed receiver integrates a two-dimensional array of optical antennas and a programmable optical processor (POP) on a silicon photonic platform. With respect to a conventional single-aperture receiver, the POP-assisted receiver recovers the complex dynamics of the optical chaos, ensuring a high degree of correlation between the transmitted signal and the received signal, even for a high degree of turbulence. Our results demonstrate the possibility of establishing and maintaining reliable, secure communication in a chaos-based crypto-system in a free space optical link of km-range length.

## Graphical abstract

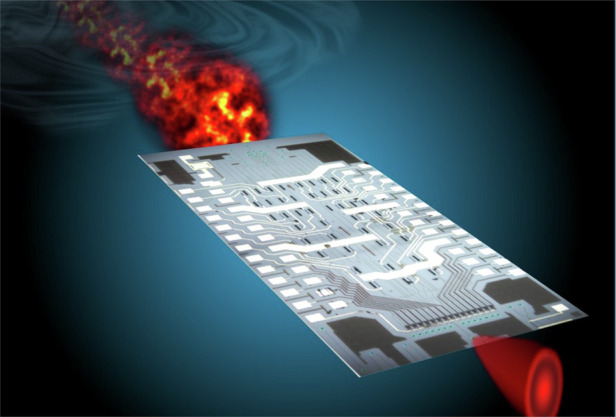

## Introduction

The emergence of Free Space Optical (FSO) communication systems marks a promising era for high-speed, cost- effective, and license-free communication, particularly for line-of-sight applications^1,2^. Unlike their radio frequency (RF) counterparts, FSO systems have many advantages, including higher bandwidth, narrower beam divergence, reduced power consumption, and lower mass requirements^3^. Moreover, FSO systems, characterized by highly directional optical beams, offer a heightened level of security compared to RF systems^4^.

Secure communications are fundamental for many sectors including banking, healthcare, and government to ensure privacy and prevent malicious eavesdropping and interference. However, the confidentiality of data transmitted through the free space might be compromised by beam spreading, especially over long distances^5^, or by beam scattering due to atmospheric particles. The security of optical transmissions can be enhanced by chaos-based techniques^6^. The field of chaos-based communication, stemming from Shannon’s pioneering work in 1948 on signals with maximal entropy^7^, has gained traction, sustained by the first successful demonstration of chaotic synchronization in 1990^8^. Characterized by their aperiodic, yet deterministic nature, chaotic signals exhibit high sensitivity to initial conditions, enabling the generation of a theoretically infinite number of low cross-correlated signals. Significant progress has been made in chaos-based communication over optical fiber links^9–11^, where the key distribution rate is 0.75 Gbit/s^10^, and the encryption data rate reaches 60 Gbit/s over 100 km of propagation^11^ and increases to 100 Gbit/s over 800 km^12^. In these systems, the practical realization of chaos-based communication is sensitive to signal distortions due to attenuation, noise, and filtering effects, which may impair the required synchronization between the transmitter and receiver. With respect to fiber communications, such effects are expected to be worsened by atmospheric turbulence in chaos-based communications FSO systems^13^. Turbulence is responsible for beam scintillation, consisting of random fluctuations of the traveling wave irradiance^14^, provoking rapid variations of the received power. Recently, some examples of chaotic FSO communications over short-link turbulent channels have been demonstrated^15,16^. However, no direct observation of the effect of turbulence on chaotic optical signals has been reported yet.

**Fig. 1:**
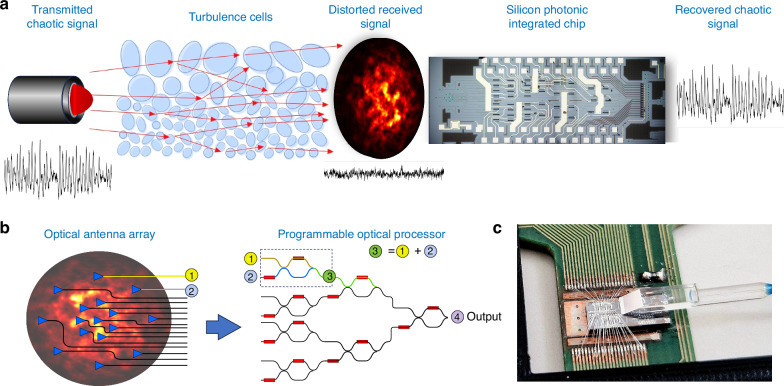
Mitigation of atmospheric turbulence effects on a chaos-based cryptosystem. **a** Turbulence induces beam spreading, wandering, and scintillation reducing the chaos complexity and compromising the system’s reliability. A programmable optical processor (POP) is used to dynamically compensate for these effects and restore chaos properties in real-time, thereby ensuring the robustness of the cryptosystem. **b** Schematic of the photonic integrated receiver, including an array of optical antennas and a self-configuring mesh of MZIs (POP). **c** Close-up photo of the Silicon photonic chip bonded to the circuit board employed for the control of the POP

In this paper, we provide a direct observation of the detrimental effects of atmospheric turbulence on the properties of chaotic optical signals, showing a substantial reduction of their chaotic dynamics. Then, as illustrated in Fig. 1a., we show that the properties of the chaotic signals propagated through a turbulent FSO link can be fully recovered by using a multi-aperture receiver assisted by a Programmable Optical Processor (POP). The POP used in this work is implemented in a silicon photonic platform and includes a self-configuring mesh of tunable Mach–Zehnder interferometers (MZIs)^17^. The multi-aperture is an on-chip optical antenna array realized with grating couplers connected to the POP (Fig. 1b). Recent results have shown the potential of reconfigurable MZI architectures for manipulating FSO beams, demonstrating the separation of spatially overlapped orthogonal FSO beams^18^, establishing optimal communication channels through arbitrary optical systems^19^, and the real-time compensation of atmospheric turbulence^20^. Here, we show that a POP-based receiver can recover the complex dynamics of chaotic signals deteriorated by transmission through a turbulent environment. System performances are evaluated by characterizing the complexity of the received chaotic beam by means of crucial figures of merit of chaos theory (i.e., correlation dimension) in several atmospheric turbulence conditions. Our results demonstrate the possibility of establishing and maintaining reliable real-time communication in a chaos-based cryptosystem, even in the presence of strong atmospheric turbulence.

## Results

### Propagation of chaotic optical beams through turbulence-free FSO links

The investigation of atmospheric turbulence effects on optical chaos requires establishing a baseline, consisting of the inspection of chaos complexity after transmission through a non-turbulent free-space system. To this end, specific tests were carried out to verify that the operation of the POP-assisted receiver does not alter the dynamics of the chaotic signal transmitted in a turbulence-free link. Details on the architecture of the employed POP and its working principle can be found in the Methods and in previous works, where it was utilized as a receiver for classical (non-chaos encrypted) FSO communications^20,21^.

**Fig. 2:**
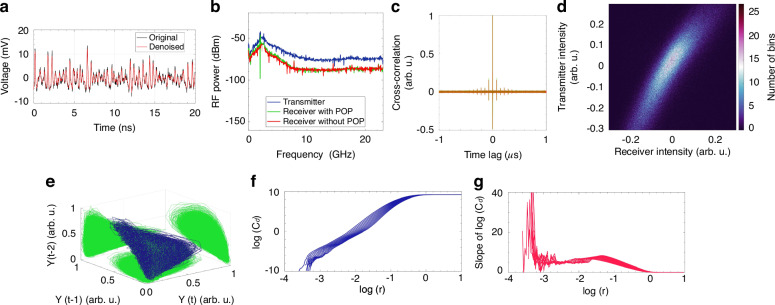
Properties of an optical chaotic signal under no turbulence effects. **a** Original (black) and wavelet denoised (red) time series of the experimental chaotic signal. **b** The RF spectrum of the transmitted chaotic signal (blue) is shown for two reception scenarios: after propagation through a non-turbulent FSO link using either a single-aperture receiver (red) or a multi-aperture receiver assisted by a POP (green). **c** 1D (normalized) cross-correlation function of the reference transmitted signal versus the signal received by single-aperture (red) or by the multi-aperture (green) receiver. **d** 2D cross-correlation function of the received intensity versus the transmitted intensity with the POP-assisted receiver (**e**) 3D phase space reconstruction of the transmitted chaotic time series (in arb. units). **f** Correlation integral C_*d*_(r) versus the sphere radius *r*. **g** Slope of the correlation integral C_*d*_(r) versus the sphere radius *r*

The chaotic signal is generated using conventional external optical feedback (EOF)^22,23^. The experimental setup is described in the Methods, while the data-denoising procedure required to analyze the chaotic signal is discussed in the Supplementary Information. A typical time trace of the signal generated by the EOF source is shown in Fig. 2a. The RF power spectra of the signal (Fig. 2b, blue curve) exhibits pronounced harmonics around 2 GHz, which are the signature of the chaotic nature of the signal. After propagation through a non-turbulent FSO link, such shape of the received spectra is preserved when the optical signal is received by either using a single-aperture receiver, consisting of a fiber-coupled collimator (red), or by a multi-aperture receiver assisted by a POP (green).

Figure 2c shows the normalized 1D cross-correlations of the transmitted signal with the signal received by the single-aperture receiver (red curve) and by the POP-assisted multi-aperture receiver (green curve). Results demonstrate that the POP does not affect correlation performance. Henceforth, in this work, such result is assumed as the baseline for the subsequent analysis in the presence of turbulence, as well as the target performance of the system (all the cross-correlation measurements are normalized to the baseline performance). The 45°-positive slope of the 2D cross-correlation in Fig. 2d further confirms the POP receiver’s nondetrimental effect. This map shows a 2D histogram of the optical intensity at the output of the POP-assisted receiver versus the transmitted intensity (the correlation computation is performed after mean-centering and normalization of the time series).

The long-term behavior of a dynamical system is graphically represented by the attractor of the system, retrieved through phase space reconstruction of the measured time series. Strange attractors, which are identified when the system exhibits complex non-repeating behavior, suggest the presence of chaotic dynamics, as observed in Fig. 2e for the optical signal generated by the EOF source. The correlation dimension *d* quantifies the intrinsic dimensionality of the attractor by quantifying the correlation of points in phase space. Typically, it is a non-integer value, and the higher its value, the higher the degree of complexity in the system’s dynamics. To obtain the correlation dimension from the measured time series, we used the correlation integral *C*_*d*_(*r*) proposed by Grassberger and Procaccia^24^ (see Fig. 2f), which provides the normalized count of points in the phase space that lie within a certain distance *r* from one another. Figure 2g represents the slope of log(*C*_*d*_(*r*)) as a function of log(*r*), allowing for the estimation of the correlation dimension *d*, which for the considered signal is ~4.87, as indicated by the plateau. A noise titration of the chaotic time series without turbulence gives a significant value for the noise limit *NL* = 80% (further details on the correlation dimension computation and the noise titration^25^ technique are provided in the Supplementary Information). Hereafter, this result is used as our baseline since it refers to a signal unaffected by turbulence.

### Turbulence effects on optical chaos

**Fig. 3:**
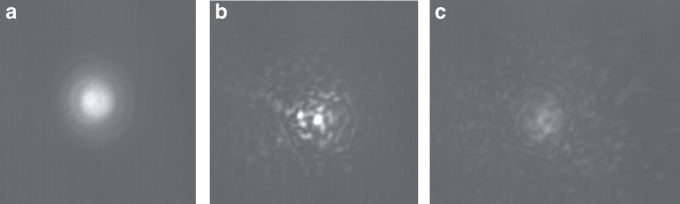
Camera images of a Gaussian-shaped chaotic optical beam propagated through a turbulent FSO link. **a** Reference beam under no turbulence conditions, and when artificial turbulence is introduced emulating an outdoor link of *L* = 100 m with refractive index parameter (**b**) *C*_n_^2^ = 10^*−*11^ m^*−*2*/*3^, and (**c**) *C*_n_^2^ = 10^*−*10^ m^*−*2*/*3^

In this section, we provide a direct observation of the detrimental effect of atmospheric turbulence on the properties of chaotic signals. In our indoor FSO link, turbulence is artificially generated using a spatial light modulator (SLM) that emulates the conditions of an outdoor FSO link with a length *L* up to 1 km and a refractive index parameter *C*_n_^2^ as large as 10^*−*9^ m^*−*2*/*3^ (see “Methods”). Figure 3 shows the cross-sectional intensity profile of a Gaussian beam, captured using a near-infrared camera before (Fig. 3a) and after (Fig. 3b, c) the SLM. In these cases, the phase screens generated by the SLM emulate the effects of turbulence for *L* = 100 m, with a structure parameter of *C*_n_ = 10^*−*11^ m^*−*2*/*3^ (Fig. 3b) and *C*_n_^2^ = 10^*−*10^ m^*−*2*/*3^ (Fig. 3c). As the turbulence magnitude increases, the Gaussian shape of the reference beam gets distorted, and exhibits pronounced speckle-like features, which are the typical signature of beam scintillation^26^. There is no significant spatial correlation between such different speckles.

**Fig. 4:**
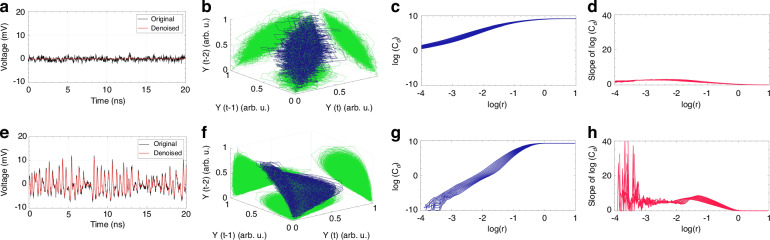
Turbulence-induced degradation of chaotic time traces and restoration with a POP-assisted receiver. Time series of the received chaotic signal (**a**) without and (**e**) with turbulence compensation performed by the POP-assisted receiver. 3D phase-space reconstruction for the received time series (**b**) without and (**f**) with turbulence compensation. Correlation integral C_*d*_(r) and relative slope of C_*d*_(r) versus sphere radius *r*, for the received time series (**c**, **d**) without and (**g**, **h**) with turbulence compensation. In these examples, a refractive index parameter *C*_n_^2^ = 10^*−*11^ m^*−*2*/*3^ for a propagation distance *L* = 100 m is considered

Figure 4a–d show the measured degradation of chaos properties when the single-aperture receiver is used. Compared to the reference signal of Fig. 2a, e, a deterministic pattern is no longer observable in the time trace of Fig. 4a, and there is no clear signature of a chaotic attractor in Fig. 4b. The same impact is observed on the correlation integral and successive estimation of its dimensionality *d* (see Fig. 4c, d). Actually, the correlation integral is hardly quantifiable due to the lack of a visible plateau, as observed in Fig. 4d (without a plateau, there is no power-law scaling with vicinity size for the attractor).

### Optical chaotic signal recovery using a POP receiver

In previous works, we demonstrated that an integrated POP receiver can mitigate the time-varying intensity fading caused by atmospheric turbulence on a classical FSO communication link^20,21^. Here, we use the POP for the first time to receive a chaotic signal degraded by turbulence-induced scintillation.

Results show that both the time trace (Fig. 4e) and the attractor shape (Fig. 4f) of the received signal look very similar to those of the reference signal in the absence of turbulence (see Fig. 2a, e). Likewise, the shape of the correlation integral C_*d*_(r) in Fig. 4g and its slope in Fig. 4h align well with the turbulence-free conditions (the correlation dimension is about 5.03). Table 1 reports the estimated correlation dimension *d* of chaotic signals affected by turbulence with refractive index parameter *C*_n_^2^ comprised between 10^*−*11^ m^*−*2*/*3^ and 10^*−*10^ m^*−*2*/*3^. As previously stated, if turbulence is not compensated by the POP, the value of the correlation distance *d* cannot be estimated. In contrast, for all the considered turbulent conditions, the compensation performed by the POP provides values of *d* ranging from 4.77 to 5.03, which are very close to the reference value of 4.87. The correlation dimension analysis is supplemented by the noise titration technique detailed in the Supplementary Information. We observe that an increase in the level of turbulence leads to a decrease in the noise limit. This implies a partial “shadowing” of chaos at the receiver due to turbulent-induced distortions on the chaotic attractor geometry, making the detection of chaos harder by the titration technique. Interestingly, when turbulence is compensated by the POP, the noise limit remains at a constant level equal to that of the non-turbulent case (i.e., *NL* = 80%) as shown in Table 1. This implies an effective elimination of the turbulence effect with a robust recovery of the optical chaotic signal.

**Table 1:** Correlation dimension *d* and noise limit *NL* for increasing C_n_^2^, retrieved for the received time series under turbulence effect, with and without compensation by the POP-based receiver

*C*_n_^2^ [m^*−*2*/*3^]	No turbulence	10^−11^		10^−10.5^		10^−10^	
		Single Apert.	POP	Single Apert.	POP	Single Apert.	POP
*d*	4.87	—	5.03	—	4.77	—	4.90
*NL* (%)	80	70	80	60	80	40	80

**Fig. 5:**
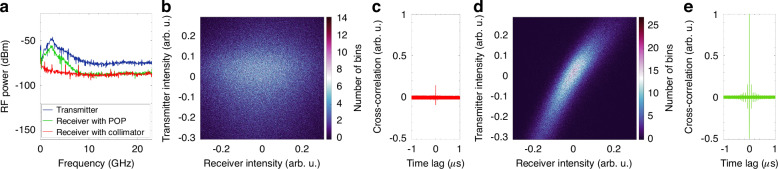
Frequency domain and correlation analysis of chaos recovery performed by the POP receiver. **a** RF spectrum of the chaotic signal at the transmitter (reference blue curve) and after propagation in the turbulent FSO link (*C*_n_^2^ = 10^*−*11^ m^*−*2*/*3^, *L* = 100 m) when a single-aperture (red) or a multi-aperture with a POP receiver (green) is used. 2D cross-correlation function of the received intensity versus the transmitted intensity, (**b**) with the single-aperture and (**d**) with the multi-aperture receiver. 1D (normalized) cross-correlation function of the received intensity versus the transmitted intensity, (**c**) with the single-aperture and (**e**) with the multi-aperture receiver

Figure 5 provides deeper insight into the effects of atmospheric turbulence on an optical chaotic signal and the restoration provided by the POP. Figure 5a shows that turbulence (*C*_n_^2^ = 10^*−*11^ m^*−*2*/*3^) significantly alters the RF power spectra of the received signal (red curve) because the low-frequency components around 2 GHz disappear entirely. Instead, such components are clearly visible in the RF spectrum of the signal recovered by the POP (green curve), a shape very similar to that of the transmitted signal (blue curve). When turbulence is not compensated, the point spread in the 2D cross-correlation of Fig. 5b confirms the lack of similarity between the transmitter and the receiver time series. Instead, when the POP receiver is used, a high degree of cross-correlation is observed, as shown by the 45°-slope feature in Fig. 5d.

Quantitatively, the effect of uncompensated turbulence turns into a significant reduction of the cross-correlation coefficient, which drops to about 0.14, with respect to our baseline (panel c); instead, the correlation performance is fully recovered when the POP receiver is used (panel e).

**Fig. 6:**
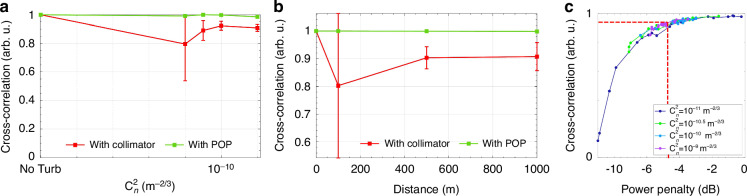
Normalized cross-correlation coefficient in different turbulence conditions. **a**, **b** Measured cross-correlation coefficient between the reference transmitted signal and the signal received by the single-aperture (red) and by the multi-aperture receiver (green) for increasing values of the structure parameter *C*_n_^2^ (*L* = 100 m, **a**) and for increasing propagation distance *L* (*C*_n_^2^ = 10^−11^ m^−2/3^, **b**). Each point provides the mean value of 20 independent measurements and the bars indicate the standard deviation. **c** Cross-correlation coefficient as a function of the power penalty of the received time series after 100 m of propagation in a turbulent environment (with increasing *C*_n_^2^), without compensation. The red line marks the critical threshold for the normalized cross-correlation at 0.9 with respect to the baseline, corresponding to approximately −4.5 dB of power penalty. The penalty is evaluated with respect to the maximum detected power (0 dB). Below the threshold, the cross-correlation drops, meaning a worsening of the system performance

Further, we investigated the performance of the system under diverse conditions of turbulence, represented by increasing values of the structure parameter *C*_n_^2^ and by increasing the propagation length *L* in the SLM turbulence emulator. Figure 6a shows the measured cross-correlation when the propagation distance is *L* = 100 m, while *C*_n_^2^ spans from 10^*−*11^ m^*−*2*/*3^ to 10^*−*9^ m^*−*2*/*3^, including the case without turbulence (SLM off). Each point is obtained by averaging the cross-correlation coefficient estimated over 20 measurements (i.e., using 20 uncorrelated phase screens generated with the same value of *C*_n_^2^), and each point is associated with the relative standard deviation (std). When the single-aperture receiver is used (red), a lower mean value and a higher standard deviation are observed with respect to the POP receiver (green). It should be noted that the worst case (*C*_n_^2^ = 10^*−*11^ m^*−*2*/*3^) is not associated with the highest turbulence degree. This can be explained by looking at the beam shapes of Fig. 3. When *C*_n_^2^ = 10^*−*11^ m^*−*2*/*3^, the size of the speckle features and their mutual spacing is comparable to the size of the receiving single aperture collimator having a diameter of 600 µm; therefore, a high-intensity fading is observed when such bright spots randomly move in the receiver plane. In contrast, for a higher degree of turbulence, the scintillated beam front is fractured into smaller and closer features, which reduce the variability in time of the power collected by the single aperture collimator. Instead, the multi-aperture receiver combines coherently the spatially uncorrelated portions of the incoming beam, resulting in a constant received power^20^. As a result, the mean correlation coefficient remains close to the reference value and its standard deviation is almost null for any degree of turbulence.

This result is confirmed by the experiment reported in Fig. 6b, where we evaluated the performance of the system as a function of the propagation distance *L* between 0 m and 1 km while keeping constant the value of *C*_n_^2^ = 10^*−*11^ m^*−*2*/*3^. Figure 6c shows that, in the case of the single-aperture receiver, the deterioration of the cross-correlation coefficient is inherently linked to the intensity fading of the received signal. Each curve corresponds to a different value of *C*_n_^2^, and each point represents the cross-correlation coefficient relative to an individual (not averaged) measurement carried out for a given configuration of the SLM. Results indicate that the cross-correlation coefficient reduces if the received power drops, and in the considered system, the maximum power penalty tolerated is only 4.5 dB.

## Discussion

In this work, we provided direct experimental evidence of the detrimental effects of propagation through a turbulent environment on the complex dynamics of chaotic optical signals. The changes in the properties of chaos complexity are inherently associated with the time-varying fading of the received power caused by atmospheric turbulence when a non-adaptive single-aperture receiver is used. Such effects may lead to synchronization loss in chaos-based secure FSO communication, thus affecting the reliability of chaos-based secure communication links^27,28^. Synchronization can be recovered by increasing the power of the transmitted signal^16^, yet this solution is not efficient in terms of power and costs. Our self-adaptive multi-aperture receiver enables the recovery of the chaos properties even in the most adverse turbulent conditions, with no power penalties with respect to the turbulence-free FSO link. In all the considered cases, the POP-assisted receiver guarantees a cross-correlation coefficient between the original chaotic signal and the signal received after propagation through the turbulent link that is equal to the reference value measured in the absence of turbulence. These results show the potential of the proposed system for implementing reliable self-synchronization of chaos-based cryptosystems in real FSO links with a length of several km. In these systems, free-space losses must be compensated by using high-gain optics in front of the photonic chip (such as arrays of lenses and/or a telescope) in such a way that the power coupled to the MZI mesh is above the sensitivity of the integrated PDs (−45 dBm) and the automated controller can operate properly.

Since the optical antennas (grating couplers) have a broad optical bandwidth (>30 nm around 1550 nm), and the mesh of balanced MZI is also broadband, interference from other FSO signals at different wavelengths may occur. This issue can be mitigated by integrating wavelength-selective filters onto the same silicon chip to isolate the spectrum of the chaotic signal while rejecting signals at other wavelengths. Notably, implementing the photonic processor did not require any customization of the fabrication process so any commercial silicon photonic foundry could produce the photonic chip.

We demonstrated the benefit of the multi-aperture adaptive receiver for a chaotic signal with carrier wavelength in the C-band, where photonic technologies for the realization of the integrated POP are mature. However, the presented approach can be applied to different spectral ranges, including the mid-infrared (mid-IR) domain. Recently, mid-IR sources have gained attention due to their favorable atmospheric transmission characteristics and resilience to environmental factors^29^. They also demonstrate chaos-based private communication over short links (tens of meters) and moderate (tens of Mbit/s) data rates^30^. Thanks to the lower sensitivity of mid-IR wavelengths to fog and turbulence effects with respect to the near-IR range^31^, using our approach in the mid-IR range could enable the realization of FSO links with even longer distances. Moreover, chaos-based encryption offers a promising alternative to quantum encryption, such as quantum key distribution systems, which is hardly implementable in the mid-IR range due to the absence of single photon detectors at these wavelengths.

Finally, using a multi-aperture photonic chip at the transmitter side would compensate for both scintillation and beam wander effects, thus increasing the end-to-end coupled power and improving the reliability of the system. As demonstrated for classical FSO communications^19^, POP-assisted pairs of multi-aperture transmitters/receivers can also be used to establish optimal orthogonal communication channels through arbitrary media, enabling the coexistence of classical and chaos-encrypted signals in the same physical link.

## Methods

### Experimental setup

**Fig. 7:**
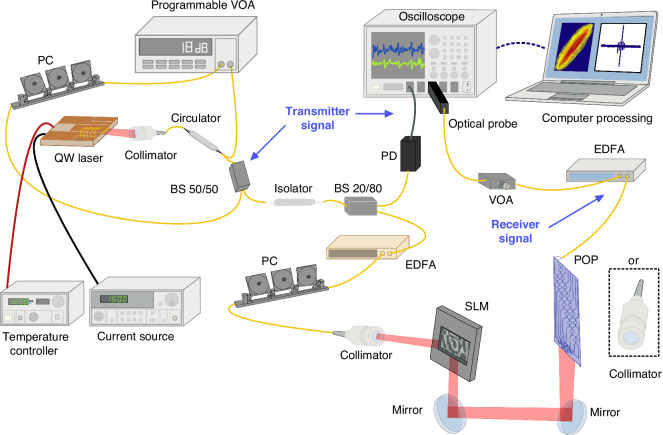
Schematic of the experimental setup. PC polarization controller, VOA variable optical attenuator, BS beam splitter, EDFA Erbium-doped fiber amplifier, SLM Spatial Light Modulator

Figure 7 shows the experimental setup employed in this work. A continuous-wave distributed-feedback Quantum-Well (QW) laser operating in single mode at 1.55 µm is biased by using a precision current source (ILX Lightwave LDX-3412), whereas its temperature is set to 293 K through a temperature controller (ILX Lightwave, LDT-5412). The laser beam is collimated into a single-mode optical fiber and is then divided into a transmission path and a feedback path by using a 50/50 fiber beam splitter. The feedback signal propagates through a 7-m-long fiber cavity before being reinjected into the laser’s active region by means of an optical circulator. A programmable variable optical attenuator (VOA, Hewlett Packard, 8156 A) is utilized to adjust the laser output power that is fed back into the cavity. A polarization controller (PC) inside the fiber cavity sets the bandwidth of the chaos. An optical isolator protects the laser from back reflections coming from the transmission path. A 20/80 splitter splits the transmitted power in such a way that 80% couples to a photodetector, while the remaining 20% is directed to the free-space optics path. In the latter, an erbium-doped fiber amplifier amplifies the transmitted signal, and a PC adjusts its polarization state. A fiber-optic collimator with a diameter of 1 mm is used to launch the light into a free-space collimated beam. The free-space path has a physical length of 3 m and includes a SLM, which is used to emulate the effects of atmospheric turbulence on outdoor links with a length of up to 1 km. To this end, arbitrary phase screens are generated by the SLM according to a Modified Von Karman Spectrum model to describe the desired atmospheric turbulence statistics^32^. Two different receivers are used to detect the signal after propagation in a free-space path: a single aperture receiver, consisting of a fiber-optic collimator with a diameter of aperture of 600 µm, or a multi-aperture receiver, consisting of a 2D array of integrated optical antennas followed by the POP. The input aperture of the POP, that is the diameter of the optical antenna array, is ~360 µm. The output power of both receivers (fiber collimator or POP) is amplified to match the dynamic range of the oscilloscope (Tektronix DPO77002SX, optical probe DPO7OE1). Data are recorded with a rate of 50 GSample/s to track the fast dynamics of the laser’s chaos.

### Integrated programmable optical processor (POP)

The integrated photonic processor was fabricated via a multi-project wafer run by a commercial silicon photonics platform (AMF, Singapore). The photonic integrated circuit is represented in Fig. 1 and consists of two stages: a 2D array of 16 grating couplers and a POP. The grating couplers sample the incoming optical beam, and the POP combines them coherently. They are arranged in outer (180 µm radius) and inner (60 µm radius) rings of respectively 8 and 7 grating couplers, and a central one. The POP is a 16 × 1 self-configuring binary mesh realized in the Silicon Photonics platform, composed of 15 balanced Mach-Zehnder interferometers (MZIs). Each one is endowed with two thermal shifters, and one of the outputs of each MZI is connected to an integrated photodiode (PD). The POP self-configures by exploiting local control loops for each MZI, needing neither prior calibration of the MZIs, nor prior knowledge of the turbulent optical system^33^. To this aim, the POP is connected through wire bonding to a custom electronic board that reads the PDs’ signal and changes the actuators’ working point. This board implements the required control loops that compensate for the relative amplitude and phase differences between the two input signals, maximizing the output power. The control finds the right power to be applied to each actuator by minimizing the received power at each PD. Finally, the output of the last MZI is coupled vertically to a single-mode fiber.

### Atmospheric turbulence emulation

A SLM is inserted in the free-space path to emulate the effects of atmospheric turbulence on the propagation of a chaotic optical beam. The SLM is software programmed to implement phase screens according to the Modified Von Karman Spectrum model^32^

1 $${\varphi }_{n}(\kappa )=0.033{C}_{n}^{2}\frac{\exp \left(-\frac{{\kappa }^{2}}{{\kappa }_{m}^{2}}\right)}{{({\kappa }^{2}+{\kappa }_{0}^{2})}^{11/6}},\qquad0\le \kappa\, <\, \infty ;\qquad{\kappa }_{0}=\frac{2\pi }{{L}_{0}};\qquad{\kappa }_{m}=\frac{5.92}{{l}_{0}}$$

where *κ* is the spatial frequency vector and *L*_0_ and *l*_0_ are the outer and inner scale of turbulence respectively. The structure parameter *C*_n_^2^ is a critical metric in characterizing atmospheric turbulence. It quantifies the magnitude of refractive index fluctuations along the optical path, offering valuable insights into the spatial distribution and intensity of turbulent eddies within the atmosphere. A higher *C*_n_^2^ value signifies more severe turbulence, resulting in greater wavefront distortions experienced by propagating light waves. In the reported experiments, we emulated conditions from moderate to strong turbulence (10^*−*11^ m^*−*2*/*3^ ≤ *C*_n_^2^ ≤ 10^*−*9^ m^*−*2*/*3^) for an FSO link of 100 m. Hence, the atmospheric fluctuations in refractive index are pronounced, leading to significant distortion of optical wavefronts. Such turbulence conditions can be implemented with a phase-only screen generated by a single SLM, as also discussed in a recent work^20^. For longer paths and/or stronger turbulence degrees, multiple phase screens are required, which can be obtained by cascading multiple SLMs^34^.

## Supplementary information

Supplementary Information: Optical Chaotic Signal Recovery in Turbulent Environments Using a Programmable Optical Processor

## Data Availability

The data that support the findings of this study are available from the corresponding author upon reasonable request.
