# Supplementary material for: Optical chaotic signal recovery in turbulent environments using a programmable optical processor
Source: Light Sci Appl. 2025 Mar 21;14:131. doi: 10.1038/s41377-025-01784-3 (PMC11928515; doi:10.1038/s41377-025-01784-3)
Supplement: Supplementary file 1 — Supplementary Information: Optical Chaotic Signal Recovery in Turbulent Environments Using a Programmable Optical Processor [file 41377_2025_1784_MOESM1_ESM.docx]

# Supplementary Information: Optical Chaotic Signal Recovery in Turbulent Environments Using a Programmable Optical Processor

**Sara Zaminga**1,***, Andres Martinez**2**, Heming Huang**1**, Damien Rontani**3**, Francesco Morichetti**2**, Andrea Melloni**2**, and Frédéric Grillot**1,4

1LTCI Te´le´com Paris, Institut Polytechnique de Paris, Palaiseau, 91120, France

2Dipartimento di Elettronica, Informazione e Bioingegneria, Politecnico di Milano, Milano, 20133, Italy

3Chair in Photonics, LMOPS UR 4423 Lab, CentraleSupe´lec & Université de Lorraine, Metz, 57070, France

4Center for High Technology Materials, University of New-Mexico, Albuquerque, NM 87106, USA

*[sara.zaminga@telecom-paris.fr](mailto:sara.zaminga@telecom-paris.fr)

^*^These authors contributed equally: Sara Zaminga, Andres Ivan Martinez.

## Analysis of chaos complexity

**Denoising** Additive white and Gaussian noise can corrupt the estimation of chaos complexity. In order to recover the original signal from the noisy data, we follow the Wavelet Transform approach[^1^](#_bookmark2). It consists in decomposing the noisy signal into groups of coefficients at different frequency levels in order to decorrelate (separate noise and useful signal) the different discrete wavelet transforms. Because the signal is contained in a small number of coefficients of such a transform, all other coefficients essentially contain noise. By filtering the coefficients relative to high- frequency components in the wavelet domain, most of the additive white and Gaussian noise is eliminated. Indeed, whereas a conventional low-pass filter uniformly removes high-frequency components that could have originated from nonlinear/chaotic behavior, wavelet denoising adaptively distinguishes between noise and meaningful signal components at different scales (multi-resolution approach). In this context, the choice of the thresholding technique is crucial to remove the noise present in the signal while preserving its characteristics. To avoid distortion and information loss, Stein’s Unbiased Risk Estimate method[^2^](#_bookmark3) and soft thresholding[^3^](#_bookmark4) are exploited. The final step consists of the computation of the corresponding inverse wavelet transform for the signal reconstruction.

**Dimension correlation analysis** Signal denoising is followed by phase space reconstruction[^4^](#_bookmark5), wherein a high- dimensional embedding of the observed time series is reconstructed to capture the dynamics of the system. Time delay embedding technique is exploited to illustrate the reconstructed state space from the time series *x*(*t*) with a delay *τ* of one and two time steps, forming a three-dimensional representation: [*x*(*t*)*, x*(*t −τ*)*, x*(*t −* 2*τ*)]. To correctly retrieve the properties of the system, the estimation of the embedding delay and embedding dimension is critical. The embedding delay is chosen where the first local zero-crossing of the auto-correlation function is identified[^5^](#_bookmark6). The optimal embedding dimension is determined according to the False Nearest Neighbour method[^6^](#_bookmark7)working as follows: if the distance between two nearby data points in the reconstructed phase-space significantly changes after increasing the embedding dimension, they are false neighbors resulting from an incomplete unfolding of the attractor. In the False Nearest Neighbour method, the embedding dimension is iteratively increased until the proportion of false neighbors saturates to a low value, usually less than 1%. For the correlation analysis, we consider typical embedding dimensions comprised between 20 and 30. The estimation of the correlation dimension is obtained according to the Grassberger-Procaccia Algorithm[^7^](#_bookmark8). It relies on the computation of an approximation of correlation integral, which quantifies the probability that points within a certain distance of each other remain close under the system’s evolution. This approximation is known as a correlation sum and is defined by:

$C_{d}\left( r \right)=\frac{1}{N^{2}}\sum_{\begin{aligned} i,j=1 \\ i\neq j \end{aligned}}^{N} \Theta(r-\left\| \boldsymbol{X}_{\boldsymbol{i}}-\boldsymbol{X}_{\boldsymbol{j}} \right\|)$ (1)

where *N* is the number of data points considered; Θ(*·*) is the Heaviside function; **X***_i,_ _j_* are two points on the reconstructed attractor embedded in a *d_E_* -dimensional phase space;
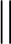
 *·
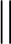
* is the Euclidean norm; and *r* is the typical radius of a local hyper-sphere defining a neighborhood centered on **X***_i_*. To extract the correlation dimension, we use the following expression

$d=\lim_{\begin{aligned} r\to0 \\ N\to+\infty\end{aligned}} \frac{\partial\log(C_{d}\left( r \right))}{\partial log(r)}$ (2)

Equation ([2](#_bookmark0)) is understood as the existence of a power-law scaling between the correlation sum and the neighboring radius, which also corresponds to a signature of self-similarity in the attractor’s geometry. In practice, we can estimate the correlation dimension by computing the slope of the logarithmic plot of *C_d_*(*r*) versus *r*, and detecting a plateau over a finite range of radii.

**Titration of chaos with noise** The titration technique is a statistical-based approach to detect the presence of chaos and nonlinear behavior in time series[^8^](#_bookmark9). It is based on the use of Wiener-Volterra series, which are nonlinear autoregressive (AR) models with the following mathematical expression

$x_{n+1}=a_{0}+\sum_{0\leq i_{1}\leq\kappa} a_{i_{1}}x_{n-i_{1}}+\sum_{0\leq i_{1}, i_{2}\leq\kappa} a_{i_{1},i_{2}}x_{n-i_{1}}x_{n-i_{2}}+\ldots+\sum_{0\leq i_{1},\ldots, i_{D}\leq\kappa} a_{i_{1},\ldots,i_{d}}x_{n-i_{1}}x_{n-i_{2}}\ldots x_{n-i_{D}}$ (3)

where *κ* is the memory depth; *D* is the maximum degree of the polynomial terms in the nonlinear AR model; *x_n_* = *x*(*n*∆*t_s_*) are samples from the initial time series with ∆*t_s_* a sub-sampled time-step; and (*a_i_*1 *, · · · , a_i_*1*,··· ,id* ) are the coefficients of the model obtained with a Gram–Schmidt algorithm applied on linear and nonlinear auto-correlation coefficients estimated from the time series[^9^](#_bookmark10). When performing the titration of chaos, the relative performance of one-time step prediction of linear AR and nonlinear AR models are compared for the time series of interest subjected to an increasing amount of additive numerical noise. The noise limit corresponds to the lowest level of noise power for which one-step prediction of a linear AR model outperforms that of a nonlinear AR model. A noise limit *>* 0 is considered as a signature of the presence of chaos. This approach is particularly suited for the detection of chaos in experimental data even with low signal-to-noise ratio and has been already applied successfully in the context of nanotechnological systems[^10^](#_bookmark11) and spintronic devices[^11^](#_bookmark12). In the titration technique, the tuple of hyper-parameters (*κ, d,* ∆*t_s_*) needs to be optimized to adjust the sensitivity of the method to the presence of chaos. This was performed on the non-turbulent (baseline) chaotic data with grid-search optimization. In our study, we have considered Volterra-Wiener series with the following hyper-parameters: *κ* = 15, *d* = 2 and ∆*t_s_* = 0*.*1 ns, corresponding to a sub-sampling factor of five with respect to the sampling period of the oscilloscope.

## Generation of chaotic nonlinear dynamics

Temporal chaos can be generated through several methods such as external optical feedback (EOF)[^12^](#_bookmark13)^,^[^13^](#_bookmark14), optical injection[^14^](#_bookmark15), and electrical modulation[^15^](#_bookmark17) of the laser bias. EOF has been chosen because of its effectiveness and prompt implementation[^16^](#_bookmark18)^,^[^17^](#_bookmark19).

As shown in Fig. 7 of our paper, the EOF setup consists of a beam splitter, a circulator, a polarization controller (PC), and a programmable variable optical attenuator (VOA). The circulator is used to reinject the feedback beam within the laser’s active region. The VOA tunes the feedback strength, defined as the ratio between the back-reflected power that couples inside the laser cavity and the total power emitted by the laser. The feedback strength can be attenuated from 60 dB (corresponding to a condition of total feedback attenuation) to 0 dB (corresponding to maximum feedback, that is 50% of the total emitted power). The PC is necessary since polarization may affect the strength and effectiveness of the feedback mechanism, impacting also the chaos bandwidth. This is crucial for the message to be enciphered while performing a chaos-based secure communication. The broader the chaos bandwidth, the higher the data rate of the enciphered message. Figures [S1](#_bookmark1)a,b show typical time trace and the frequency spectrum of the chaotic signal generated in our setup.

In order to determine the optimal working conditions for an eventual secure communication, the 1D Auto- Correlation function is estimated. The autocorrelation of a chaotic time series shows some distinct features due to its complex dynamics. Indeed, in chaotic systems, the auto-correlation function tends to decay rapidly as the lag increases, exhibiting a narrow peak at zero time lag, as observed in Fig. [S1](#_bookmark1)c. Despite the overall decay, Fig. [S1](#_bookmark1)c displays also periodic peaks at time lags corresponding approximately to multiples of 14 MHz frequency. These peaks are due to the 7-m length of the external cavity. The presence of periodic or quasi-periodic behavior embedded within the chaotic dynamics implies regular patterns or repeating structures in the chaotic attractor, that can be exploited by adversaries to predict the future states of the system, compromising the unpredictability required for secure encryption. To mitigate this risk, the feedback strength is set around 0.8% (18 dB of attenuation) as a compromise between optical spectrum broadening (used as an indicator of chaos complexity) and minimization of the amplitude of the satellite peaks in the
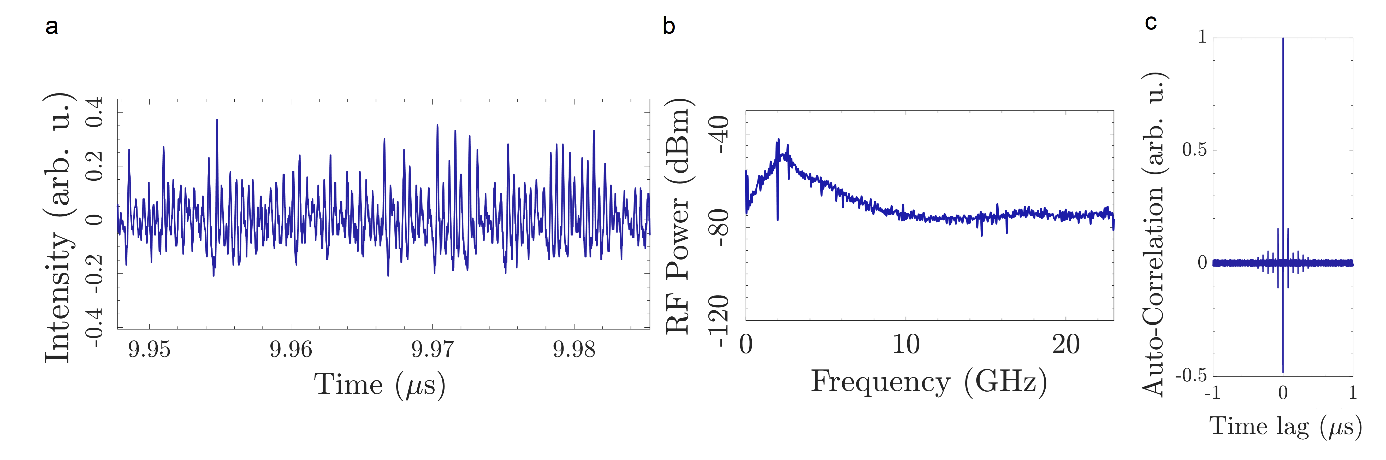
autocorrelation function.

**Figure S1. Properties of chaotic nonlinear dynamics. a.** Time series of the transmitted chaotic signal. **b.** RF spectrum of the transmitted chaotic signal. **c.** 1-D auto-correlation function of the transmitted chaotic signal.

# References

1. Farge, M. Wavelet transforms and their applications to turbulence. *Annual Review of Fluid Mechanics* **24**, 395–458 (1992).
2. Donoho, D. L. & Johnstone, I. M. Adapting to unknown smoothness via wavelet shrinkage. *Journal of the* *American Statistical Association* **90**, 1200–1224 (1995).
3. Donoho, D. L. De-noising by soft-thresholding. *IEEE transactions on information theory* **41**, 613–627 (1995).
4. Takens, F. Detecting strange attractors in turbulence. *Dynamical Systems and Turbulence, Warwick 1980* 366–381 (2016).
5. Kantz, H. & Schreiber, T. *Nonlinear Time Series Analysis* (Cambridge University Press, Cambridge, New York, 2004).
6. Kennel, M. B., Brown, R. & Abarbanel, H. D. I. Determining embedding dimension for phase-space reconstruction using a geometrical construction. *Physical Review A* **45**, 3403–3411, DOI: <10.1103/PhysRevA.45.3403> (1992).
7. Grassberger, P. & Procaccia, I. Characterization of strange attractors. *Physical review letters* **50**, 346 (1983).
8. Poon, C.-S. & Barahona, M. Titration of chaos with added noise. *Proceedings of the National Academy of* *Sciences of the United States of America* **98**, 7107–7112 (2001).
9. Barahona, M. & Poon, C.-S. Detection of nonlinear dynamics in short noisy time series. *Nature* **381**, 215–217 (1996).
10. Hu, S. Q. & Raman, A. Chaos in atomic force microscopy. *Physical Review Letters* **96**, 036107 (2006).
11. Devolder, T. *et al.* Chaos in magnetic nanocontact vortex oscillators. *Physical Review Letters* **123**, 147701 (2019).
12. Peil, M. *et al.* Routes to chaos and multiple time scale dynamics in broadband bandpass nonlinear delay electro-optic oscillators. *Physical Review E* **79**, 026208, DOI: <10.1103/PhysRevE.79.026208> (2009).
13. Romeira, B. *et al.* Broadband chaotic signals and breather oscillations in an optoelectronic oscilla- tor incorporating a microwave photonic filter. *Journal of Lightwave Technology* **32**, 3933–3942, DOI: <10.1109/JLT.2014.2308261> (2014).
14. Simpson, T. B., Liu, J. M., Gavrielides, A., Kovanis, V. & Alsing, P. M. Period-doubling cascades and chaos in a semiconductor laser with optical injection. *Physical Review A* **51**, 4181–4185, DOI: <10.1103/PhysRevA.51.4181> (1995).
15. Hori, Y., Serizawa, H. & Sato, H. Chaos in a directly modulated semiconductor laser. *Journal of the Optical* *Society of America* **5**, 1128–1133, DOI: <10.1364/JOSAB.5.001128> (1988).
16. Ohtsubo, J. *Semiconductor lasers: stability, instability and chaos*, vol. 111 (Springer, 2012).
17. Fischer, I., Hess, O., Elsä*β* er, W. & Göbel, E. High-dimensional chaotic dynamics of an external cavity semiconductor laser. *Physical review letters* **73**, 2188 (1994).
